# Time-resolved absolute measurements by electro-optic effect of giant electromagnetic pulses due to laser-plasma interaction in nanosecond regime

**DOI:** 10.1038/srep27889

**Published:** 2016-06-15

**Authors:** F. Consoli, R. De Angelis, L. Duvillaret, P. L. Andreoli, M. Cipriani, G. Cristofari, G. Di Giorgio, F. Ingenito, C. Verona

**Affiliations:** 1 ENEA - C.R. Frascati - Dipartimento FSN, Via E. Fermi 45, 00044 Frascati, Italy; 2 Kapteos, Alpespace - bât. Cleanspace 354 voie Magellan, 73800 Sainte-Hélène du Lac, France; 3 INFN–Università di Roma “Tor Vergata”, Via del Politecnico 1, 00133 Roma, Italy

## Abstract

We describe the first electro-optical absolute measurements of electromagnetic pulses (EMPs) generated by laser-plasma interaction in nanosecond regime. Laser intensities are inertial-confinement-fusion (ICF) relevant and wavelength is 1054 nm. These are the first direct EMP amplitude measurements with the detector rather close and in direct view of the plasma. A maximum field of 261 kV/m was measured, two orders of magnitude higher than previous measurements by conductive probes on nanosecond regime lasers with much higher energy. The analysis of measurements and of particle-in-cell simulations indicates that signals match the emission of charged particles detected in the same experiment, and suggests that anisotropic particle emission from target, X-ray photoionization and charge implantation on surfaces directly exposed to plasma, could be important EMP contributions. Significant information achieved on EMP features and sources is crucial for future plants of laser-plasma acceleration and inertial-confinement-fusion and for the use as effective plasma diagnostics. It also opens to remarkable applications of laser-plasma interaction as intense source of RF-microwaves for studies on materials and devices, EMP-radiation-hardening and electromagnetic compatibility. The demonstrated extreme effectivity of electric-fields detection in laser-plasma context by electro-optic effect, leads to great potential for characterization of laser-plasma interaction and generated Terahertz radiation.

Interactions between high energy and high intensity lasers with matter produce particle flux and electromagnetic radiation over a wide range of energy^1^,^2^. The generation of transient fields of very high intensity in the radiofrequency-microwave regime has been observed for femtosecond to nanosecond laser pulses with 10^11^–10^20^ W/cm^2^ intensity, on both conductive and dielectric targets^3^,^4^,^5^,^6^,^7^,^8^,^9^,^10^,^11^,^12^,^13^,^14^,^15^,^16^,^17^,^18^,^19^,^20^,^21^. These fields have a bandwidth of several GHz and last for hundreds of nanoseconds. They often cause saturation and damage to the electronic equipment inside and near the experimental chamber, but on the other hand can also become an effective diagnostic tool. For these reasons, the absolute characterization of EMPs is of great importance and nowadays a very hot topic for present and future plants for laser-plasma acceleration (*PETAL*^22^, *ELI*^23^,^24^,…) and for inertial-confinement-fusion (*NIF*^6^,^12^,^13^, *LMJ*^22^,…). Once the sources will be well understood, laser-plasma interaction could be used also as powerful generator of transient RF-microwave fields, volumetrically distributed with respect to the point of emission, for many applications as interaction with materials and devices, EMP-radiation-hardening and electromagnetic compatibility studies.

EMPs have been previously characterized with conductive probes^3^,^4^,^5^,^6^,^7^,^8^,^9^,^10^,^11^,^12^,^13^,^14^,^15^,^16^,^17^,^18^,^19^,^20^,^21^, but only a few works specifically address the absolute measurements of the associated electric fields, which are accomplished by *D-dot* and *B-dot* probes (sensitive to 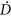 and 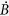 respectively)^25^,^26^. This is mainly due to the difficulties in performing reliable measurements with suitable signal-to-noise ratio (SNR). The conductive structure of these probes usually determines low measurement accuracy in near-field characterization^27^. This is a major problem in experimental chambers for laser-plasma-interaction (LPI) experiments, normally filled with metallic equipment, and sets a limit on the probe minimum distance from the interaction locus. Conductive probes cannot be put in direct view of the plasma, because parasitic currents can be induced on them by ionizing radiation emitted from LPI (charged particles, and UVs-Xrays by photoelectric effect), and can propagate to the oscilloscope heavily affecting the E-field measurement. EMPs may further induce spurious currents on the external conductor of the coaxial cables connecting probes to oscilloscope. Moreover, time integration is required to get electric fields from 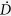 measurements; this greatly amplifies the low-frequency noise (where important part of the signal is), and requires suitable filtering^6^. 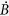 measurements are even worst, since plane-wave approximation (inappropriate inside a conductive chamber) is used. In this work we describe the first time-resolved direct measurements, using the linear electro-optic (Pockels) effect in crystals^28^, of single vectorial components of EMP electric fields due to laser-plasma interactions on nanosecond regime, at laser intensities typical of inertial-confinement-fusion. This allowed us to perform, for the first time, measurements with the detector in direct view of the plasma and rather close to it.

Dielectric electro-optic (EO) probes have allowed reliable measurement of transient electric fields^29^,^30^,^31^,^32^,^33^,^34^,^35^, with many advantages versus classical conductive probes, in terms of field vectorial selectivity (one component measurement rejecting the orthogonal ones), bandwidth, dynamics, temporal and spatial resolution, low dimensions and invasiveness^32^,^33^,^34^,^36^,^37^. Indeed, their effective permittivity may induce a local perturbation on fields measured in vacuum. This remains much lower and localized than for conductive probes^32^,^33^,^34^ and with offline calibration can be estimated for de-embedding. The dielectric structure insures no parasitic currents even in these harsh environments. Their intrinsic low sensitivity is generally not detrimental when dealing with high intensity field measurements, as for EMPs. So far, they have been used in air, water, and for atmospheric-pressure plasma jet sources^32^,^34^,^38^,^39^,^40^. Here we report about their application in vacuum and specifically in LPI.

## Methodology

### Electro-optic probe

**Figure 1:**
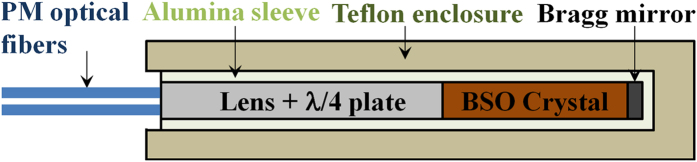
Scheme of the EO-probe.

Figure 1 shows the EO-probe structure^32^,^41^. Measurements of external electric fields are performed by detecting the change of polarization state, induced by electro-optic effect, of a continuous-wave laser probing beam having *λ*_p_ = 1550 nm and circular polarization (obtained with the λ/4 plate), propagating in a <111>-cut 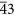 m Bi_12_SiO_20_ (BSO) crystal of 5 mm length. This is the *polarization state modulation* technique^29^,^30^,^31^,^32^,^33^,^34^,^42^,^43^. BSO is intrinsically isotropic, becoming here birefringent because of the 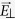 component of the external field, orthogonal to the laser wave-vector 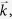 which is parallel to the <111> direction of the crystal. Actually, in our probe 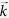 has been slightly misaligned with this direction, so that light coming from the first fiber can be collected by the second, after total reflection on the dielectric Bragg mirror. This double-passage increases the sensitivity, and simplifies the probe mounting close to plasma. Elliptical polarization on the output probe beam is caused by the generated anisotropy; information on electric field is contained on: (1) induced dephasing between the two linearly-polarized components of the elliptical polarization 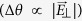, (2) orientation of eigendielectric axes with respect to the 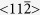 optical axis: *ξ*_±_ = π (3 ± 1)/4 − *α*_*E*_/2, being *α*_*E*_ the angle between 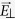 and that axis^31^,^42^,^43^. In particular, it is 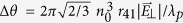
43, where *r*_41_ and *n*_0_ are the Electro-Optical coefficient of BSO crystal and its refractive index in absence of applied external E-field, respectively. At *λ*_*p*_ wavelength *n*_0_ = 2.405 and *r*_41_ has been measured for known external electric fields in the (10 Hz–2 GHz) range, as reported in ref. 44. As a brief summary of these measurements, at low frequencies (below 100 kHz) it resulted *r*_41_ = 5.4 ± 0.4 pm/V and at higher ones (above 10 MHz) *r*_41_ = 4.7 ± 0.2 pm/V, with more complex behaviour in the (100 kHz–10 MHz) range. Thus, by using a wave polarization analyzer - reported in detail in refs 42 and 43 −Δ*θ* and *ξ*_±_ can be measured and from them the two components of 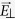 can be simultaneously determined^29^,^30^,^31^,^32^,^33^,^34^,^42^,^43^. The laser probe beam has a double passage in the crystal and the electric-field is then averaged over a τ = 80 ps time frame (flying time of photons through 10 mm of BSO crystal). From this, it is possible to achieve the probe cutoff frequency^29^,^32^: *f*_*c*_ = 0.443/τ = 5.5 GHz.

Kapteos^TM^ built a custom version of the EOP-P2R02-BS050 probe to adapt it to vacuum conditions used in the experimental chamber of ABC facility^45^,^46^. A polarization maintaining (PM) fiber is joined to the set of lens +λ/4 plate, followed by crystal and mirror (Fig. 1). An Alumina sleeve (30 mm length and 4 mm diameter) contains the whole structure. The custom probe was then enclosed in a 3 mm thick Teflon shield, having 4 mm and 10 mm internal and external diameters, respectively, and protecting it from direct X-ray radiation coming from plasma. Shield permittivity *ε*_*r-Teflon*_ ~ 2.1 leads to partial impedance matching of the probe (effective permittivity *ε*_*r-eff*_ ~ 9^31^,^32^,^33^,^34^) with surrounding vacuum, increasing the sensitivity. The overall configuration and the small bandwidth, centered at 1550 nm, of the detection system (tailored infrared fast photodiodes, PM fibers and optics) ensures effective rejection of main laser (λ_0_ = 1054 nm) stray light, and of 400–700 nm light emission from scintillation of BSO by X-rays^47^,^48^,^49^. All components are dielectric and non-magnetic, with ultra-low loss tangent; the probe is insensitive to magnetic fields up to more than 3 T^36^, three orders higher than those expected in this experiment in the probe position. Optical fiber connection allowed the electronics to be placed suitably far from experiment, eliminating their free-space-coupling with EMP. The sensitivity of complete setup was preserved by using two tailored vacuum optical-feedthroughs with minimum insertion loss. The unit eoSense HF-2A-09L provided laser probe generation, monitored probe sensitivity along time, executed demodulation and determination of the *E*_*X’*_ and *E*_*Y’*_ components of 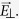 Electro-optic effect occurs on femtosecond time-scales, leading to intrinsic bandwidths exceeding 10 THz, with *f*_*min*_ in the kilohertz range^32^. Indeed, system bandwidths are limited by round-trip time of laser through the crystal (*f*_*c*_) and electronics frequency cutoffs. To improve the SNR, dedicated low-noise-amplifiers were used before the 3.5 GHz Lecroy-735Zi oscilloscope, and this determined *f*_*max*_ ~ 0.5 GHz.

The electro-optic probe was offline calibrated using a Transverse ElectroMagnetic (TEM) cell^50^,^51^. This is a closed waveguide having 50 Ω characteristic impedance, with core basically constituted by parallel conductive plates where a TEM electromagnetic wave is propagating. A uniform electric field was thus applied to the electro-optic probe placed in the centre of the cell for calibration purposes. The field strength is simply given by the potential difference between the plates divided by the distance separating them, and in our case anyway previously calibrated. The modulus of the scattering parameter *S*_11_ (reflection coefficient) of the TEM cell was verified to be lower than −20 dB when used with a 50 Ω termination load, with or without the presence of the EO-probe inside. It means that the traveling-wave regime was insured when calibrating the electro-optic probe, and local wave-reflections due to the BSO/Alumina/Teflon/air interfaces did not modify remarkably the electric field at small distances from the probe. This guaranteed a correct and precise calibration procedure of the EO-probe up to the cutoff frequency of the TEM cell (6 GHz), providing accurate data on orientation of the crystal axes, measurement dynamics (>120 dB Hz^0.5^), intrinsic sensitivity (<20 kV/m for single shot pulses), vectorial selectivity (>40 dB) and spatial resolution (<5 mm), whereas the field sign was still undetermined. The calibrated probe is thus able to measure external electric fields with ±30% accuracy, much better than for classical conductive probes. The system is intrinsically stable for temperature variation and this was verified during the LPI experiments.

## Results

### Experimental measurements

**Figure 2:**
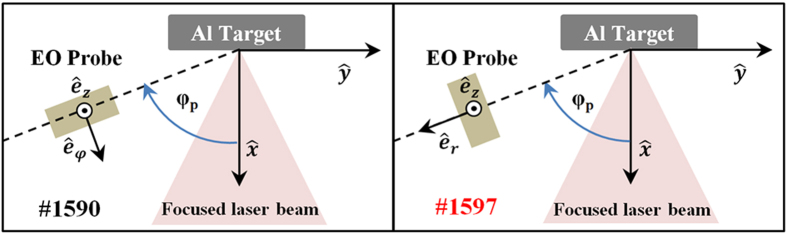
Scheme of the experiment in the two configurations represented by the shots #1590 and #1597.

**Figure 3:**
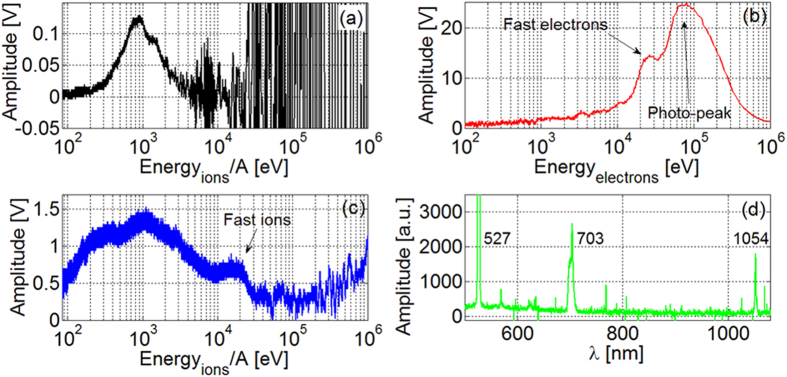
Measurement of particles and of visible light spectrum. (**a**) Faraday-cup at 53°; diamond detector at 65°: (**b**) electron and (**c**) ion energy domain; (**d**) Optical spectrometer. A = atomic mass number.

Experiments have been performed with ABC laser, a Nd:phosphate-glass nanosecond facility^45^,^46^. One circularly polarized beam of 20–30 J, with *FWHM* ~ 3 ns, fundamental wavelength λ_0_ and 10^-5^ contrast, was focused by a F/1 lens up to 50 μm diameter, leading to ~(0.3–0.5) PW/cm^2^ intensity, for normal incidence on a thick (1710–1790 mm) Al target with ~40 mm^2^ plain surface (Fig. 2). For each shot the LPI was monitored by a large number of diagnostics^45^,^46^. Thermal ion emission from plasma was measured by Time-of-Flight (TOF) detection with a set of faraday-cups; a particle contribution with *E*_*ion*_/*A* ~ 1 keV (*A* = atomic number) is shown in Fig. 3a at *φ* = 53° from the target normal. As evident, strong coupling with EMP oscillations conceals detection of particles with *E*_*ion*_/*A* ≥ 4 keV. The problem is overcome with TOF monocrystalline diamond detectors^52^,^53^, fabricated by Università di Tor Vergata with microstrip surface-interdigital connections and high hardness to EMPs. In Fig. 3b signal measured by a diamond placed at *φ* = 65° is shown in the electron-energy domain. The huge peak on the right is due to X-rays; on its left falling edge a clear indication of fast electrons is present, with ~26 keV peak energy and ~40% FWHM. Part of this hot electron population is due to resonant absorption^1^,^54^,^55^, because of highly focused intensity (even higher in some regions because of self-focusing), large focusing angle (F/1 lens) and laser circular polarization. The remaining population is due to two-plasmon-decay instability^1^,^54^,^56^, also generating the 2λ_0_/3 = 703 nm harmonic^54^,^56^. In Fig. 3d the visible light spectrum detected by the Ocean Optics HR4000 spectrometer is shown, with the clear indication of components at λ_0_, 2λ_0_/3, and λ_0_/2 (527 nm). Hot electrons escaping from plasma create an electrostatic Debye sheath which accelerates ions^2^. On the same diamond signal the trace of ~20 keV fast protons (hydrogen is always present on target surfaces as impurity^2^) was in fact detected (Fig. 3c), together with the 1 keV thermal peak measured by the faraday-cup.

**Figure 4:**
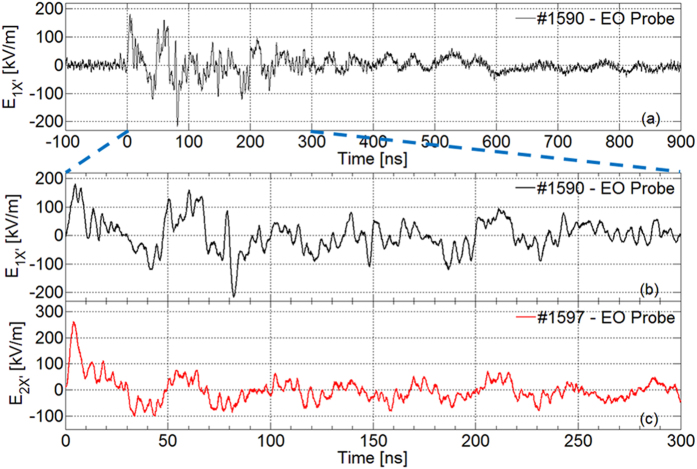
Measured *E*_*X′*_ field component for shot #1590 (**a,b**, in two different time scales) and #1597 (**c**).

**Figure 5:**
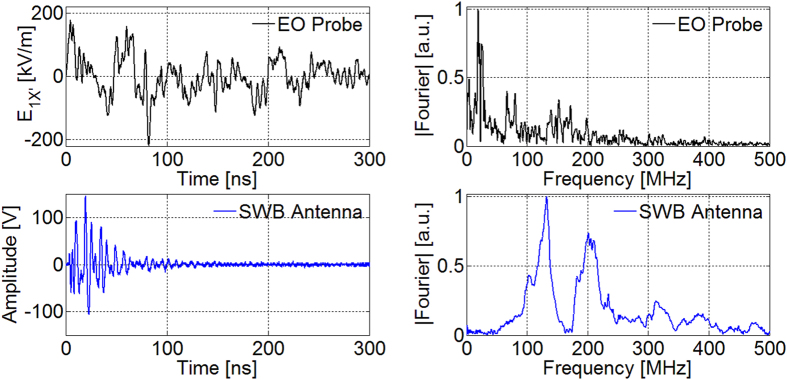
Time and frequency domain of signals of EO-probe and SWB antenna for shot #1590.

We performed two series of measurements, both with EO-probe in direct view of the target and at 85 mm distance. For the first series the probe was mounted on the *xy* plane, as in configuration indicated for shot #1590 in Fig. 2, with *φ*_*p*_ = 70°, longitudinal axis parallel to 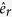 in cylindrical coordinates and Bragg mirror toward the target. In the whole campaign we were able to measure the 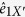 component of 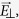 but not the 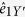 one, because of technical reasons: lower sensitivity to this component and increased background noise of the associated channel amplifier. In particular, it was (Fig. 2) 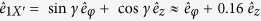, being *γ* = 81° ± 3°. In Fig. 4a,b the measured *E*_*1X′*_ component is shown for shot #1590 of this first series, in two different time scales; the axis origin was chosen at the beginning of the first intense peak. The signal appears to be constituted by two spectral components, as also shown in Fig. 5. The main contribution lasts for the total signal duration (~1 μs) and concerns frequencies below ~50 MHz. The second affects only the first 300 ns and contains higher frequencies. Maxima higher than 100 kV/m are present during the first 250 ns. Background noise is visible for *t* < 0, leading to very good SNRs for each shot. In the whole campaign it was not possible to determine the absolute field phase (i.e. sign). For this #1590 shot, a first high ‘positive’ peak is present (*FWHM* = 6.7 ns) and then a ‘negative’ large peak at ~40 ns. Thus, a rather sharp one is at ~80 ns, where the field reaches its maximum value: 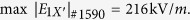 From plane-wave approximation and on the hypothesis of isotropic emission with respect to target, the overall EMP energy is thus estimated ~0.46 J, ~1.8% of the laser one.

A superwideband (SWB) antenna was also used at ~48 cm from the target^14^,^15^,^16^,^57^, in a region of the chamber close to the surface and well protected against direct plasma radiation by thick conductive objects. Results in time and frequency domain are compared in Fig. 5 with those from EO-probe. The SWB signal duration is lower, whereas its main spectral contributions near ~130 MHz and ~200 MHz find correspondence in the EO signal. The high-pass behaviour of the antenna was expected because it is sensitive to the time derivative of the electric field and because of its 50 Ω impedance matching, worst at the smallest frequencies^57^. Moreover, the antenna is placed in a section of the vacuum chamber having transversal dimension much lower than the chamber radius. So, only the EMP components at higher frequency can reasonably penetrate that small space. The antenna is also close enough to the chamber surface, where quartz windows and many vacuum radiofrequency-feedthroughs are present. As observed in references^15^,^16^, these are source of coupling of EMPs to the outside of the vacuum chamber. This energy leakage causes local electromagnetic modes excited in this section of the chamber to have low Q values, and then related fall-time behaviour faster than for modes excited in the chamber core^15^,^16^. We could suppose that the spectral content of the EO signal for *f* < 50 MHz, not revealed by the SWB antenna, might be due to direct plasma radiation (X-rays and particles), exciting these components only in the chamber core.

In the second series of measurements, probe was rotated of 90° clockwise with respect to the *z* axis (shot #1597 in Fig. 2), so 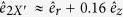. In Fig. 4c the related *E*_*2X′*_ component is shown. Presence and time-duration of the two spectral contributions observed for shot #1590 are confirmed here, too. There is still a high sharp ‘positive’ peak (correspondent to that of shot #1590) having *FWHM* = 5.4 ns, leading to 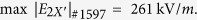 Then, the field slowly decreases, changing sign at ~30 ns. There are some large oscillations, decreasing below 100 keV/m after ~80 ns from the main peak. The time evolution for *t* > 300 ns is similar to shot #1590, and then not reported. In this case the EMP energy is ~0.54 J, ~2.2% of laser one.

### Particle-in-cell simulations

**Figure 6:**
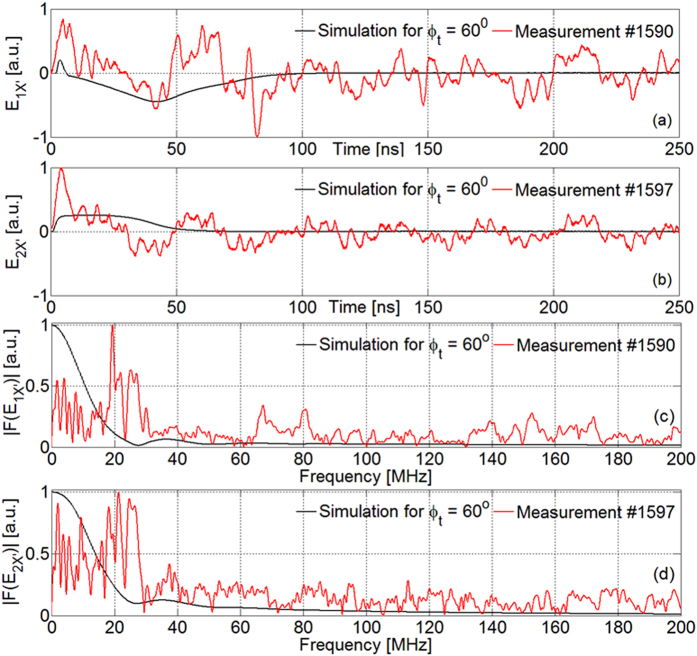
Measurement of *E*_*1X′*_ in shot #1590 with related simulations for *φ*_*t*_ = 60°: (**a**) time domain and (**c**) Fourier spectrum. The same for *E*_*2X′*_ in shot #1597: (**b**) time domain and (**d**) Fourier spectrum.

To have better insight on possible origins of signals observed we performed simplified particle-in-cell (PIC) simulations of the experiment, by CST Particle Studio solver. Space-charge effects were considered, together with secondary electron emission from Teflon^58^ and superficial charge deposition on surfaces. Considering Fig. 2, the simulated box dimensions were 150 mm, 400 mm and 200 mm along *x*, *y* and *z* axes, respectively. Absorbing boundary conditions (free-space) were set on each box surface and adaptive meshing refinement was performed for a total number of about 254000 mesh cells. Minimum and maximum mesh step size were 0.85 mm and 5.15 mm respectively, and a time step of 2.2 ps was used. The target surface was source of conical particle flows, uniform within their angle of emission *φ*_*t*_ to the target normal. We considered a Gaussian-shaped electron bunch with σ = 3 ns, 26 keV of peak energy, and 40% energy spread, as estimated from diamond measurements (Fig. 3b). An equal and synchronized bunch of protons was added, modeling the fast-ion component. In Fig. 6a,b we compare, in time domain, EO-probe measurements with results of simulations for *φ*_*t*_ = 60°. The first peak of measured *E*_*1X′*_ can be effectively associated with the simulated fast-electron peak, whereas simulated fast ions can be associated with the following measurement decrease, having minimum at ~40 ns. Later oscillations might be associated with quasi-neutral thermal components, not considered in these preliminary simple calculations. In Fig. 6c,d the same comparison is shown for the related Fourier spectra. The simulated configuration is very simple, and does not allow to get a remarkable fitting with experimental data; however, calculations indicate that the low-frequency components of measured spectra are compatible with fields generated by particle flows. Furthermore, this might confirm that the classical high-frequency components, also showed in the spectrum of the SWB-antenna signal (see Fig. 5), are likely cavity modes excited within the experimental chamber^4^,^15^,^16^.

**Figure 7:**
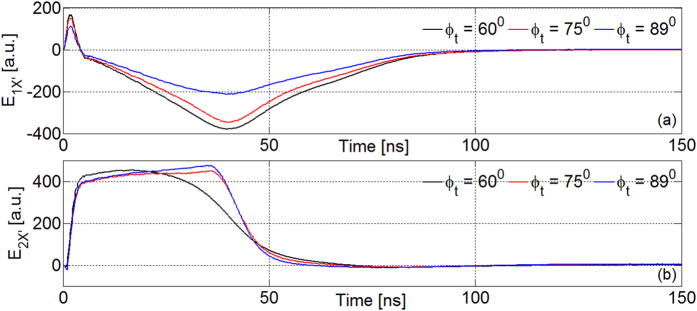
Simulations for *E*_*1X′*_ (**a**) and *E*_*2X′*_ (**b**) for different *φ*_*t*_.

Future and more accurate modelings of the experiment have to consider photoionization due to X-rays from plasma, generating a cloud of cold electrons around the external surface of the Teflon. This is expected to create a pulsed electric field, rather synchronous with the peak due to fast electrons. Effects due to charge implantation on Teflon have to be taken carefully into account, too. These phenomena help to explain the absence of the first peak of measured *E*_*2X′*_ in these preliminary simulations and the higher intensity of measured *E*_*1X′*_ peak. In Fig. 7 we show simulations of *E*_*1−2X′*_ versus *φ*_*t*_ angle; when *φ*_*t*_ > 70° particles hit the probe. *E*_*1X′*_ (~*E*_*φ*_) decreases with *φ*_*t*_ and this is due to anisotropic particle emission from target with respect to the probe. Indeed, *E*_*φ*_ became negligible in simulations with the EO-probe on the symmetry axis of the particle flow. On the other hand, *E*_*2X′*_ (~*E*_*r*_) is not particularly affected by this (Fig. 7b).

## Conclusions

We have described the first direct electric-field measurements of EMPs generated by laser-target interaction in ICF regime, performed by dielectric EO-probes directly viewing the plasma. A maximum electric field of 261 kV/m was measured, about two orders of magnitude higher than previous measurements in nanosecond lasers with much higher energy (LULI, OMEGA and NIF^6^,^8^,^10^,^12^,^13^,^18^,^20^,^21^), and comparable with maximum values reached with picosecond lasers^18^,^20^,^21^.

The analysis of measurements and preliminary simulations indicates that EMP signals are compatible with the emission of TOF-detected charged particles^10^. It also shows that EMP should be affected by anisotropic particle emission from target, X-ray photoionization and charge implantation on surfaces directly exposed to plasma. In experiments with a femtosecond laser of ~100 mJ (with moderate plasma ionizing-radiation) the main EMP contribution resulted from neutralization-currents flowing through the target holder^18^,^21^. In nanosecond facilities high intensity neutralization-currents were measured^59^, but it has been shown that their contribution to EMP is small^18^,^21^. Figure 5 suggests that here this could be associated with the main spectral components ~130 MHz and ~200 MHz detected by both EO-probe and SWB antenna.

The performed measurements allowed to get important information on laser-plasma interaction. Future experiments of this type and tailored numerical studies will be important for the better understanding of EMP sources in different regimes, key point of future plants for laser-plasma acceleration and for inertial-confinement-fusion, as well as for the use of EMPs as effective plasma diagnostics. This also opens the significant application of laser-plasma interaction as powerful source of transient RF-microwave fields of high intensity, volumetrically distributed with respect to the point of emission, for interaction with materials and devices, EMP-radiation-hardening and electromagnetic compatibility studies. Moreover the demonstration that electro-optic effect can be an extremely effective method for detecting electric fields in laser-plasma context, leads to a great potential for characterization of the intrinsic LPI and of the generated Terahertz radiation.

## Additional Information

**How to cite this article**: Consoli, F. *et al*. Time-resolved absolute measurements by electro-optic effect of giant electromagnetic pulses due to laser-plasma interaction in nanosecond regime. *Sci. Rep*. **6**, 27889; doi: 10.1038/srep27889 (2016).
